# Diagnosis of bilateral pharyngoesophageal diverticula with using swallow contrast-enhanced ultrasound: a case report

**DOI:** 10.3389/fonc.2024.1351509

**Published:** 2024-08-14

**Authors:** Xue Wan, Hongyan Liu, Juxian Liu, Yulan Peng

**Affiliations:** Department of Ultrasound, West China Hospital of Sichuan University, Chengdu, Sichuan, China

**Keywords:** pharyngoesophageal diverticula, diagnosis, contrast-enhanced ultrasound, thyroid nodule, case report

## Abstract

Pharyngoesophageal diverticulum (PED) is a rare disease of the esophagus that is usually asymptomatic and often found incidentally during a thyroid ultrasound examination. Due to its anatomical location close to the thyroid, it is easily misdiagnosed as a thyroid nodule, which leads to unnecessary thyroid biopsies and surgical treatment. The occurrence of a single esophageal diverticula is common, while the existence of multiple diverticula is rare. Left side diverticula are more common than right sided ones, while bilateral occurrences are rarely reported. We report an extremely rare case of bilateral pharyngeal esophageal diverticula. The patient was a 55-year-old asymptomatic man who came to our hospital after thyroid nodules were identified in another hospital. Due to the extensive clinical experience of the ultrasound physician at our facility, the patient was suspected to have bilateral esophageal diverticula, which was confirmed by using swallow contrast-enhanced ultrasound (CEUS). Consequently, unnecessary thyroid treatments were avoided in this patient. This study shows that although bilateral pharyngeal diverticula are unusual, the possibility of their existence should be considered if nodules are located posterior to the bilateral thyroid glands and have suspicious imaging characteristics. Particular attention should be given to nodules located on the right side of the thyroid, which are sometimes overlooked easily due to their very low incidence. If real-time ultrasound cannot be used in making the diagnosis, PED can be further identified using swallowing CEUS to avoid unnecessary thyroid fine needle aspiration (FNA) and surgical treatment.

## 1 Introduction

An esophageal diverticulum (ED) is a relatively rare disease of the esophagus (1, 2). ED is a localized swelling of one or all layers of the esophageal wall, forming a pouch that communicates with the esophageal cavity (1). According to the site of the diverticula, ED can be categorized as pharyngoesophageal diverticula (PED), mid-esophageal diverticula, and epiphrenic diverticula (3). Most patients are asymptomatic; however, a few patients may experience dysphagia, food reflux, aspiration pneumonia, weight loss, ozostomia, and cough due to food particles being retained within the diverticulum (1, 4). Anatomically, PED are located adjacent to the thyroid gland; hence, they can be easily misdiagnosed as thyroid nodules during a thyroid ultrasound examination. Moreover, due to  the presence of gas in an esophageal diverticulum, ultrasound images would show hyperechogenic areas, which is sometimes easily confused with calcification hyperechoes. If the understanding of the location and the ultrasonic image of an esophageal diverticulum is insufficient, it is easy to misdiagnose it as a thyroid malignant tumor and perform thyroid-related surgical treatments. Therefore, it is very important to pay close attention to the position of a pharyngoesophageal diverticulum and its diagnostic features on ultrasonography.

Most patients are diagnosed using a barium esophagogram, cervicothoracic CT, or endoscopy (1). Swallow contrast-enhanced ultrasound (swallow-CEUS) is another effective diagnostic method (5, 6), but it is not commonly used in the clinical diagnosis of PED. Because microbubble ultrasound contrast agents are usually administered intravenously, and the extravascular approach is seldom used, except for vesicoureteral reflux imaging (7) and uterotubography (8). Swallow-CEUS imaging involves the imaging of the digestive tract after an extravascular administration of ultrasound contrast agents. This case was diagnosed using this method, which makes this report an interesting and educational one.

In addition, cases of a single esophageal diverticulum in the pharynx are more common than cases of multiple divertciula. Furthermore, most of them are adjacent to the left lobe of the thyroid gland, while only a few are adjacent to the right lobe of the thyroid gland (9, 10). The occurrence of bilateral diverticula in the esophagopharynx is rarely reported in the literature. Herein, we report such a very rare case.

## 2 Case description

**Figure 1:**
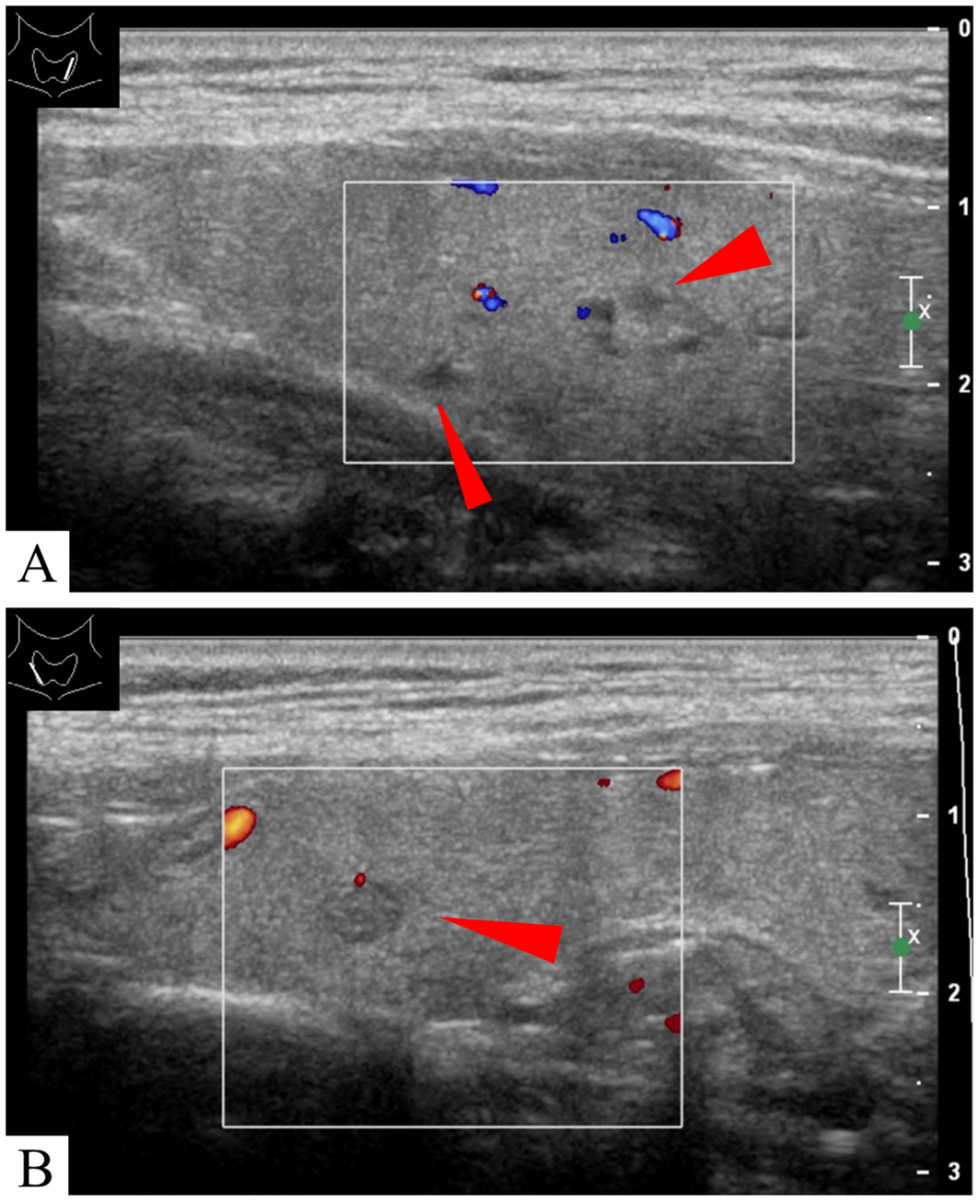
The images showed several nodules in the bilateral lobes of the thyroid (red arrows) **(A, B)**.

**Figure 2:**
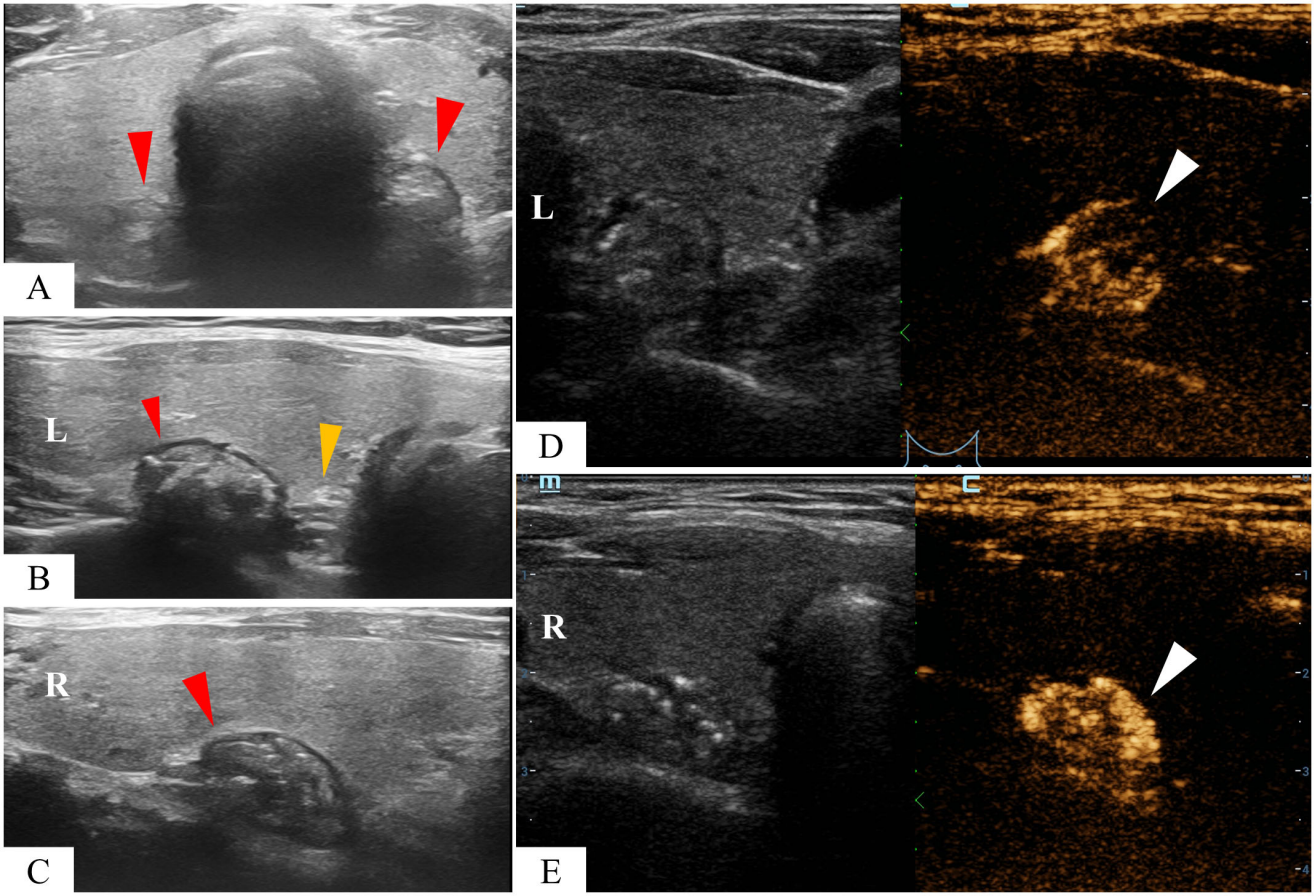
A mixed echogencitiy nodule was found behind the left and right thyroid lobes respectively (red arrowhead) **(A–C)**. The nodule on the left is abutting the esophagus (yellow arrowhead) **(B)**. Contrast agents were found in both nodules after swallowing SonoVue (white arrowhead) **(D, E)**.

**Figure 3:**
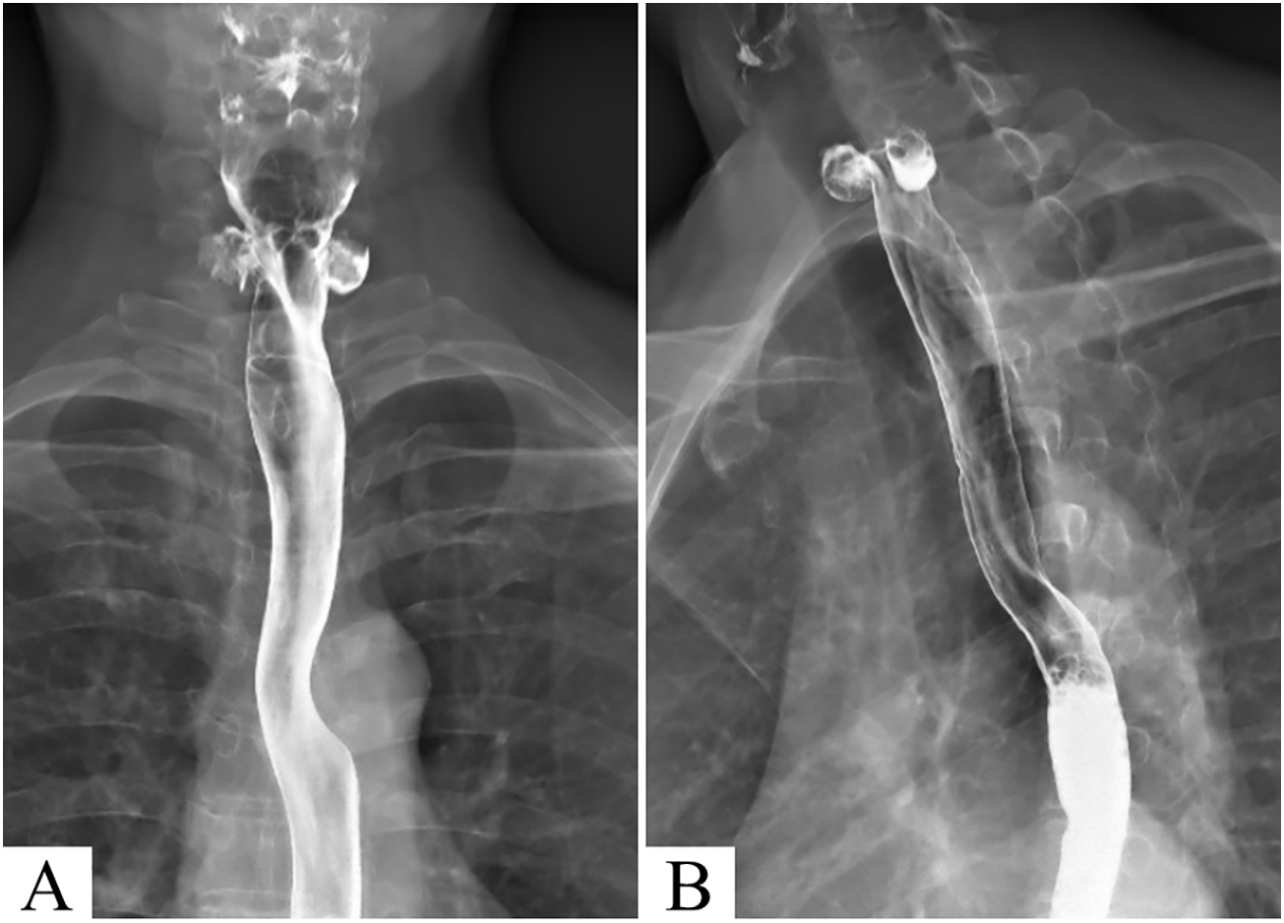
Barium esophagogram images showed two diverticula in the pharyngeal esophagus **(A, B)**.

A 55-year-old asymptomatic male patient was referred to our hospital for further treatment after thyroid nodules were identified during a physical examination in another hospital. Ultrasonography showed that in addition to nodules in the thyroid gland (multiple nodules in the lobes bilaterally, measuring 2–6 mm, with low echo or mixed echoes) (**Figures 1A, B**), there were mixed echo nodules posterior to the inferior part of the thyroid glands bilaterally (**Figures 2A–C**). The right nodule measured 16 × 10 × 16 mm, while the left nodule was 17 × 10 × 12 mm. The echoes of the two nodules were similar, both were oval, and contained strong spot-like echoes. The anterior edge of the nodules was clear, and the posterior margin of the left nodule was continuous with the wall of the esophagus (**Figure 2B**). When the patient was asked to swallow, the internal echo of the left nodule changed, and there was relative movement between the nodule and the thyroid gland. However, these features of the nodules on the right side were not obvious. According to the above characteristics, we still considered the possibility of double esophageal diverticula. To confirm the diagnosis, the patient underwent swallow-CEUS (1 mL of SonoVue diluted with about 500 mL of drinking water). The patient was instructed to swallow the contrast agent, which was then found to be present in both nodules, confirming a diagnosis of bilateral PED (**Figures 2D, E**). This patient had no discomfort; therefore, no definitive treatment was instituted; however, we opted for regular observations and follow-up. The patient has been followed up for 2 years without any discomfort, and there were no significant changes in the size of both esophageal diverticula under ultrasound examination. During the follow-up, the patient underwent a barium esophagogram, which again confirmed bilateral PED (**Figures 3A, B**).

## 3 Discussion

ED is a relatively rare benign disease of the esophagus (9, 11, 12). PED is the most common type of ED, and accounts for over 70% of all cases of ED (3). They can occur at any age but are usually diagnosed in older patients and are more common in men than in women (1). PED is classified into three types: Zenker’s diverticulum (ZD), Killian–Jamieson diverticulum (KJD), and Laimer’s diverticulum (LD), based on the different anatomic weak areas they originate from (12). ZD originates from the Killian triangle, located in the posterior midline of the hypopharyngeal and cervical esophageal junction, with the upper part bounded by the pharyngeal contractile muscle and the lower part bounded by the cricopharyngeal muscle (11, 12). An abnormal increase in pressure in the pharyngeal cavity may lead to the formation of a diverticulum in this triangular area (11). ZD is the most common type of PED, first described by the German pathologist Friedrich Albert von Zenker in 1867 (9, 13). KJD originates in the Killian-Jamieson area, located on the anterior lateral wall of the proximal esophagus, which is an anatomical area where the recurrent laryngeal nerve enters the pharynx with low resistance. This area was first described by Killian in 1908 and confirmed by Jamieson in 1934 (14). KJD is a rare type of PED (14). The incidence ratio of KJD and ZD is 1:4 (9, 14). The two can be identified and differentiated using an esophagogram because the cricopharyngeal muscle, as a diagnostic marker, shows a filling defect on imaging (15). The opening of a KJD is below the defect, while the opening of a ZD is above the defect (15). However, it is sometimes difficult to differentiate between ZD and KJD preoperatively (12). LD originates from the Laimer–Haeckerman’s triangle, which is only covered by the annular muscle on the side of the cervical esophagus, located between the longitudinal and cricopharyngeal muscles of the esophagus (12, 16). LD is also an extremely rare type of PED. The three types of PED have similar symptoms and similar treatments, and they are collectively known as PED (3). In most cases, patients are asymptomatic (11). Typical symptoms include dysphagia, reflux, halitosis, and voice changes (11). Some of the clinical manifestations of ED complications include cough due to food retention in the diverticular sac, aspiration, and even aspiration pneumonia (11). Asymptomatic patients do not need treatment and can be followed up. In this report, the patient did not have any discomfort; therefore, regular follow-up and observation were selected. For patients with symptoms, surgical and endoscopic treatment options can be considered (1). Surgical treatment options include diverticulotomy, diverticulopexy, diverticuloinversion, combined or non-combined myotomy, and myotomy alone (17). Endoscopic treatment involves a minimally invasive myotomy and removal of the diverticulum under a soft or rigid endoscope (18). If the patient is not a candidate for surgery, management through dietary changes is also recommended (1).

PED are usually solitary, with most of them occurring behind the left lobe of the thyroid gland (9); cases on the right side are unusual and rarely reported (19). Additionally, bilateral PED are very infrequent and rarely reported in the literature. Therefore, when a bilateral PED occurs, the nodules located behind the right side of the thyroid gland are more likely to be misdiagnosed as thyroid nodules than those located behind the left lobe. Our case report reminds sonographers of the need to consider the possibility of bilateral PED.

Reports show that as PED is close to the thyroid, it occasionally mimics thyroid nodules, and may be misdiagnosed as thyroid nodules, especially as thyroid malignancies, resulting in the performance of FNA or surgical treatment (20–23). The acoustic image of PED also has specific characteristics. According to the echo intensity in the diverticulum, Bai et al. divided nodules into four types: solid nodular diverticulum, gas-containing nodular diverticulum, liquid-containing nodular diverticulum, and atypical diverticular changes (9). In our case, the diverticula appeared as solid nodules, which are more easily confused with thyroid nodules. The nodules contained strong echoes, which were similar to the calcifications seen in papillary thyroid carcinoma. Generally, if a nodule identified on ultrasonography is located posterior to the thyroid gland, the possibility of a PED should be considered and verified. Possible identification methods include the following:

1. Morphologically, PED is usually oval or circular with mixed echoes inside (6).
2. The anterior edge of the focus is usually hemicyclic with hypoechoes (esophageal wall) (24). A careful inspection will show that it continues with the esophageal wall (9).
3. There is no blood flow signal in the lesion, and a little punctated blood flow can be seen in the hypoechoic wall (6).
4. There is no or slight movement of the lesion during swallowing, or the movement is out of sync with that of the thyroid gland (6, 24).
5. When drinking water, the size of the nodules changes, and there are signs of liquid and gas flow between the esophagus and the mass as the liquid or gas enters the esophagus (9). However, malignant nodules of the thyroid appear on ultrasound as distinct hypoechoic nodules with regular or irregular edges and punctured blood flow (9).

When the above methods are not obvious, oral ultrasound along with contrast agent examination can be considered. A 2015 study by Cui et al. confirmed that oral ultrasound contrast agent examination is a simple, safe, and acceptable method (5). They found that the contrast agent stayed in the ZD for more than 3 min, allowing enough time to scan the ZD and its surrounding structures. Another study reported that swallowing CEUS is a quick and sensible way to diagnose ZD before thyroid nodule ablation therapy, which aids in avoiding misdiagnosing ZD as a thyroid nodule and preventing subsequent invasive surgery (6). After using swallow CEUS, the inflow and outflow of contrast agents in the PED can be clearly observed. This is due to the continuity between the diverticulum and the wall of the esophagus as well as the connectivity between the diverticulum and the esophageal cavity. Contrast infusion showed high enhancement in PED, while no contrast infusion was observed in thyroid nodules. In this case, during swallow CEUS examination, high enhancement was observed in the nodules located behind the bilateral thyroid lobes, confirming a diagnosis of bilateral PED.

Currently, barium esophagram is considered the gold standard diagnostic method (25). ZD classification and size determination can also be performed (26). However, swallow-CEUS examination has its own unique advantages of being radiation-free and available at the bedside (5); furthermore, it can be used to identify small diverticula that esophagography may fail to identify (6). When ZD is suspected on routine ultrasound, swallowing CEUS may be the preferred method to confirm a PED diagnosis (5). When esophageal barium meal tests are negative for suspected small diverticular nodules, swallowing CEUS can be used as a supplementary diagnostic tool.

## 4 Conclusion

PED is a rare benign lesion of the esophagus, and bilateral pharyngeal diverticula are even rarer. Our case report highlights the need for sonographers to consider the possibility of pharyngeal diverticula when deep thyroid nodules are identified during examinations, regardless of which side the nodule is located. Imaging features should also be carefully identified and distinguished from thyroid nodules. If a real-time ultrasound diagnosis is difficult, further identification can be made using swallowing CEUS to avoid unnecessary thyroid FNA and surgical treatment.

## Data Availability

The original contributions presented in the study are included in the article/supplementary material. Further inquiries can be directed to the corresponding author.

## References

[1] Yam J Baldwin D Ahmad SA. Esophageal Diverticula. Treasure Island (FL: Statpearls (2023). 30422453

[2] Hussain T Maurer JT Lang S Stuck BA. Pathophysiology, diagnosis and treatment of Zenker's diverticulum. Hno. (2017) 65:167–76. doi: 10.1007/s00106-016-0302-z 27933354

[3] Strong AT Ponsky JL. Esophageal diverticula. In: Zundel N Melvin WS Patti MG Camacho D, editors. Benign Esophageal Disease: Modern Surgical Approaches and Techniques. Springer International Publishing, Cham (2021). p. 173–210.

[4] Little RE Bock JM. Pharyngoesophageal diverticuli: Diagnosis, incidence and management. Curr Opin Otolaryngol Head Neck Surg. (2016) 24:500–4. doi: 10.1097/MOO.0000000000000309 27636983

[5] Cui XW Ignee A Baum U Dietrich CF. Feasibility and usefulness of using swallow contrast-enhanced ultrasound to diagnose Zenker's diverticulum: preliminary results. Ultrasound Med Biol. (2015) 41:975–81. doi: 10.1016/j.ultrasmedbio.2014.12.003 25701519

[6] Huang HJ Chen R Sheng JG Cao KK Zhao JQ. Characteristic analysis of Zenker's diverticulum incidentally detected on multimodal neck ultrasound. J Med Ultrason (2001). (2020) 47:279–85. doi: 10.1007/s10396-019-00992-w 31848772

[7] Darge K. Voiding urosonography with ultrasound contrast agents for the diagnosis of vesicoureteric reflux in children. I Procedure Pediatr Radiol. (2008) 38:40–53. doi: 10.1007/s00247-007-0529-7 17618429 PMC2292498

[8] Exacoustos C Di Giovanni A Szabolcs B Romeo V Romanini ME Luciano D. Automated three-dimensional coded contrast imaging hysterosalpingo-contrast sonography: Feasibility in office tubal patency testing. Ultrasound Obstet Gynecol. (2013) 41:328–35. doi: 10.1002/uog.11200 22648792

[9] Bai Z Wang X Zhang Z. Pharyngoesophageal diverticulum mimicking thyroid nodules: Some interesting ultrasonographic signs. Front Oncol. (2023) 13:1030014. doi: 10.3389/fonc.2023.1030014 36824141 PMC9941519

[10] Marcy PY Benisvy D Poissonnet G Sadoul JL Thariat J. Zenker's diverticulum: The diagnostic power of ultrasound. Thyroid. (2010) 20:1317–8. doi: 10.1089/thy.2010.0140 20932178

[11] Constantin A Constantinoiu S Achim F Socea B Costea DO Predescu D. Esophageal diverticula: from diagnosis to therapeutic management-narrative review. J Thorac Dis. (2023) 15:759–79. doi: 10.21037/jtd PMC9992562 36910058

[12] Watanabe Y Taniyama Y Koseki K Ishida H Ozawa Y Okamoto H. Distinguishing Killian-Jamieson diverticulum from Zenker's diverticulum. Surg Case Rep. (2023) 9:21. doi: 10.1186/s40792-023-01599-7 36759360 PMC9911565

[13] Walts AE Braunstein G. Fine-needle aspiration of a paraesophageal diverticulum masquerading as a thyroid nodule. Diagn Cytopathol. (2006) 34:843–5. doi: 10.1002/(ISSN)1097-0339 17115442

[14] Bonacchi G Seghieri M Becciolini M. Killian-Jamieson diverticulum: Real-time sonographic findings. J Ultrasound. (2016) 19:295–8. doi: 10.1007/s40477-016-0208-3 PMC5126011 27965721

[15] Rubesin SE Levine MS. Killian-Jamieson diverticula: Radiographic findings in 16 patients. AJR Am J Roentgenol. (2001) 177:85–9. doi: 10.2214/ajr.177.1.1770085 11418403

[16] Ujiie N Taniyama Y Sato C Kamei T. Surgical intervention for Laimer's diverticulum, a rare type of pharyngoesophageal diverticulum: A case report. OTO Open. (2019) 3:2473974x19847670. doi: 10.1177/2473974X19847670 PMC6737866 31535071

[17] Costantini M Zaninotto G Rizzetto C Narne S Ancona E. Oesophageal diverticula. Best Pract Res Clin Gastroenterol. (2004) 18:3–17. doi: 10.1016/S1521-6918(03)00105-7 15123081

[18] Khullar OV Shroff SR Sakaria SS Force SD. Midesophageal pulsion diverticulum resulting from hypercontractile (Jackhammer) esophagus. Ann Thorac Surg. (2017) 103:e127–e9. doi: 10.1016/j.athoracsur.2016.07.030 28109370

[19] Kim DC Hwang JJ Lee WS Lee SA Kim YH Chee HK. Surgical treatment of Killian-Jamieson diverticulum. Korean J Thorac Cardiovasc Surg. (2012) 45:272–4. doi: 10.5090/kjtcs.2012.45.4.272 PMC3413838 22880178

[20] Achille G Castellana M Russo S Montepara M Giagulli VA Triggiani V. Zenker diverticulum: A potential pitfall in thyroid ultrasound evaluation: A case report and systematic review of literature. Endocr Metab Immune Disord Drug Targets. (2019) 19:95–9. doi: 10.2174/1871530318666180910122003 30198446

[21] Cao L Ge J Zhao D Lei S. Killian-Jamieson diverticulum mimicking a calcified thyroid nodule on ultrasonography: A case report and literature review. Oncol Lett. (2016) 12:2742–5. doi: 10.3892/ol.2016.4984 PMC5038824 27698850

[22] Nauschuetz KK Ogden LL Starling CE Saleh MJ Golding AC Traweek ST. Pharyngoesophageal diverticula simulating thyroid nodules: An unusual occurrence with unique features. Diagn Cytopathol. (2018) 46:193–7. doi: 10.1002/dc.23817 28925594

[23] Chen HC Chang KM Su WK. Incidental pharyngoesophageal diverticulum mistaken for a thyroid nodule: Report of two cases. Diagn Cytopathol. (2019) 47:503–6. doi: 10.1002/dc.24144 30632292

[24] Chen X Liu JF Gu CJ Ding SJ Zhou SX Chen XY. Ultrasonographic characteristics of Killian-Jamieson diverticula. J Clin Ultrasound. (2021) 49:527–32. doi: 10.1002/jcu.23011 33786835

[25] Chang CY Scher RL. Barium esophagogram of a Zenker's diverticulopexy. Ear Nose Throat J. (2006) 85:230, 2. doi: 10.1177/014556130608500411 16696356

[26] Vogelsang A Schumacher B Neuhaus H. Therapy of Zenker's diverticulum. Dtsch Arztebl Int. (2008) 105:120–6. doi: 10.3238/arztebl.2008.0120 PMC2696720 19633762

